# Supplementary material for: Analysis of the off-target interaction of amyloid PET tracers with human brain sulfotransferases
Source: Neurol Sci. 2026 Sep 19;47(10):819. doi: 10.1007/s10072-026-09431-4 (PMC13624043; doi:10.1007/s10072-026-09431-4)
Supplement: Supplementary file 1 [file 10072_2026_9431_MOESM7_ESM.docx]

**Supplementary table 1. Statistical analysis of RMSD trajectories for alternative docking poses.** The table summarizes the RMSD parameters calculated over 300 ns molecular dynamics simulations for PiB, flutemetamol, and flutafuranol in complex with the SULT1E1, SULT1A1, and SULT1A3 isoforms. For each ligand-protein complex, two alternative docking poses (indicated as 1a/1b, 2a/2b, or 3a/3b) are compared. The reported values include the mean of RMSD (Mean, Å), the standard deviation (SD, Å), and the slope of the linear regression (Slope) derived from the RMSD vs. time plots. These descriptors were employed to evaluate the structural convergence and stability of each binding mode.
